# Techniques of orthotopic renal transplantation in pigs. One donor to two recipients via inverted grafting

**DOI:** 10.1590/ACB360208

**Published:** 2021-02-22

**Authors:** Yoshitaka Kinoshita, Daiki Iwami, Tetsuya Fujimura, Haruki Kume, Takashi Yokoo, Eiji Kobayashi

**Affiliations:** 1 MD. Jichi Medical University – Department of Urology – Division of Renal Surgery and Transplantation – Tochigi, Japan. Jichi Medical University – Department of Urology – Division of Urology – Tochigi, Japan.; 2 MD, PhD. Jichi Medical University – Department of Urology – Division of Renal Surgery and Transplantation – Tochigi, Japan.; 3.MD, PhD. Jichi Medical University – Department of Urology – Division of Urology – Tochigi, Japan.; 4 MD, PhD. The University of Tokyo – Graduate School of Medicine – Department of Urology – Tokyo, Japan.; 5 MD, PhD. The Jikei University School of Medicine – Department of Internal Medicine – Division of Nephrology and Hypertension – Tokyo, Japan.; 6 MD, PhD. The Jikei University School of Medicine – Department of Kidney Regenerative Medicine; Keio University School of Medicine – Department of Organ Fabrication – Tokyo, Japan.

**Keywords:** Kidney, Transplantation, Surgery, Swine

## Abstract

**Purpose:**

Although transplanting two kidneys from a single donor to two recipients has some advantages, the right and left kidneys are not anatomically identical; thus, a surgical procedure considering the anatomical features of the donor kidneys is needed when transplanting them into the opposite renal fossae. Based on vast experience, the surgical details of pig orthotopic kidney transplantation from one donor to two recipients was reported.

**Methods:**

When the right kidney was transplanted to the left renal fossa, the graft was inverted upside down, not backwards, thus ensuring that the anteroposterior relationship of the renal vessels was maintained and anatomically natural vascular anastomosis could be performed.

**Results:**

Using this technique, we could have developed a pig experimental model that is safe and has a high success rate, even for researchers in the middle of their training. This technique of inverting the graft upside down was reported in human kidney transplantation to make vascular anastomosis easier.

**Conclusions:**

In pig orthotopic kidney transplantation from one donor to two recipients, an anatomically natural vascular anastomosis could be performed via inverted grafting when the right kidney was transplanted into the left renal fossa.

## Introduction

The similarity in size, anatomy and physiology between pigs and humans has made pigs the most preferred animal for organ transplantation research and training^1^. For many decades, various models have been reported for pig kidney transplantation^2^; recently, orthotopic kidney transplantation models of pigs have been used for the training of laparoscopic or robotic kidney transplantation^3^^,^ 4.

In humans, donated kidneys are generally transplanted into the iliac fossa using a retroperitoneal approach. Orthotopic transplantation is used only in cases where the iliac vessels are not suitable for anastomosis, such as in children with an inferior vena cava obstruction^5^^,^ 6. Conversely, because the iliac vessels of pigs are too narrow to anastomose, the kidney is normally orthotopically transplanted using an intraperitoneal approach. The donor’s renal artery is anastomosed to the recipient’s aorta in an end-to-side fashion using Carrel patch technique, while the donor’s renal vein is anastomosed to the recipient’s inferior vena cava in an end-to-side fashion, or renal vein in an end-to-end fashion. The donor’s ureter is anastomosed to the recipient’s ureter or bladder^2^.

The kidney was orthotopically transplanted into the left renal fossa in our facility, as the risk of the anastomosed renal artery oppressing the crossing inferior vena cava when being transplanted into the right renal fossa was considered. However, the right kidney may be the donor kidney, requiring the utmost care considering its anatomical features^7^. Therefore, the surgical procedure of pig orthotopic kidney transplantation from one donor to two recipients was demonstrated via inverted grafting.

## Methods

### Experimental animals and ethics

This experiment was approved by the IVTeC Animal Welfare Committee (permit number: IVT20-64) and was performed in the facility of IVTeC Co., Ltd. (Hyogo, Japan). Female pigs aged 3 months and weighing 20–30 kg were used. Animals were treated in accordance with the guidelines for proper conduct of animal experiments^8^.

### Anesthesia

The pigs were housed in cages under temperature and light-controlled conditions (12-hour light/dark cycle) and were freely provided with food and water. The pigs were fasted for 12 h prior to surgery, with free access to water. Sedation with an intramuscular injection comprising a mixture of ketamine (10 mg/kg), xylazine (2.0 mg/kg) and atropine (0.5 mg/body) was followed by anesthetic induction with 5% inhalational isoflurane and 3.0 L/min oxygen. After endotracheal intubation, anesthesia was maintained with 1–3% inhalational isoflurane; although spontaneous respiration is usually retained, mechanical ventilation was used according to the depth of anesthesia. A urinary catheter was placed into the bladder and lactated Ringer’s solution was dripped at 60 mL/h (adjusted according to the vital signs during the surgery) through an intravenous line placed in the auricular vein.

### Donor procedure

**Figure 1:**
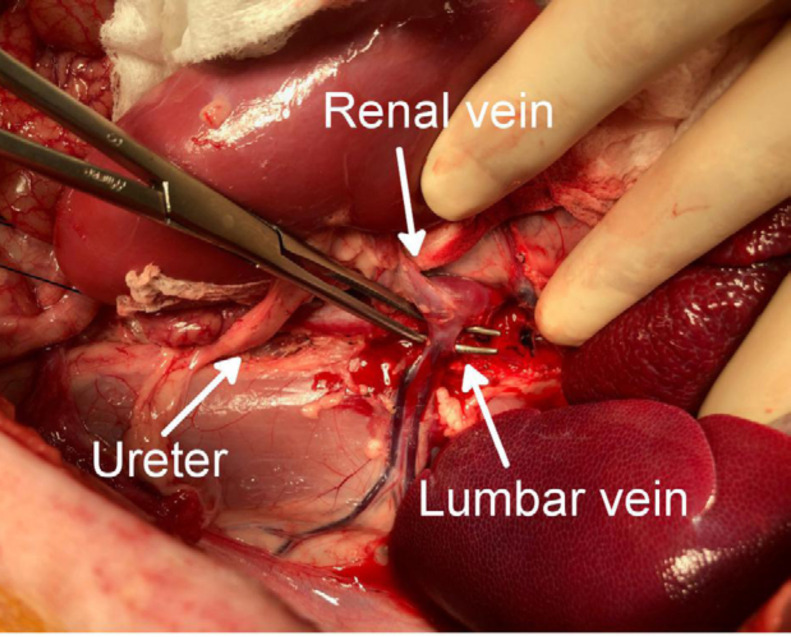
Ligation and division of the lumbar vein.

**Figure 2:**
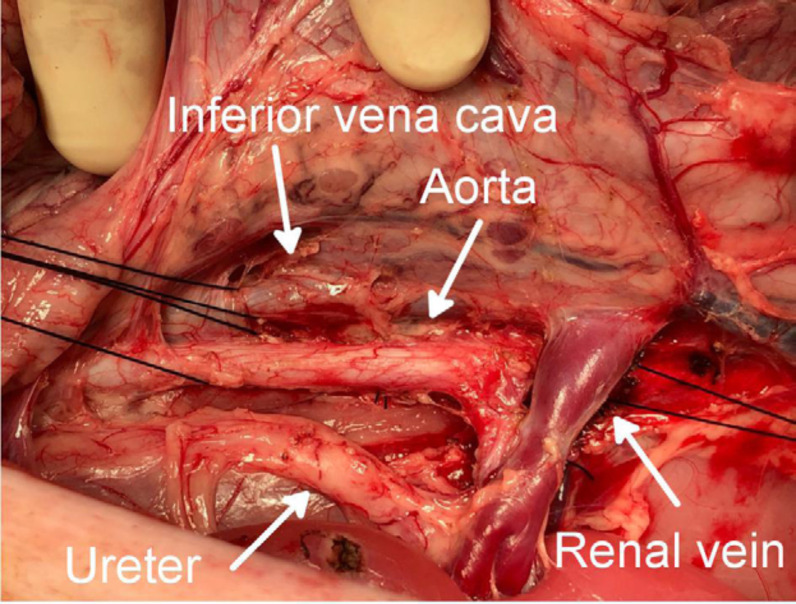
Dissection of the aorta and the inferior venacava for cannulation.

**Figure 3:**
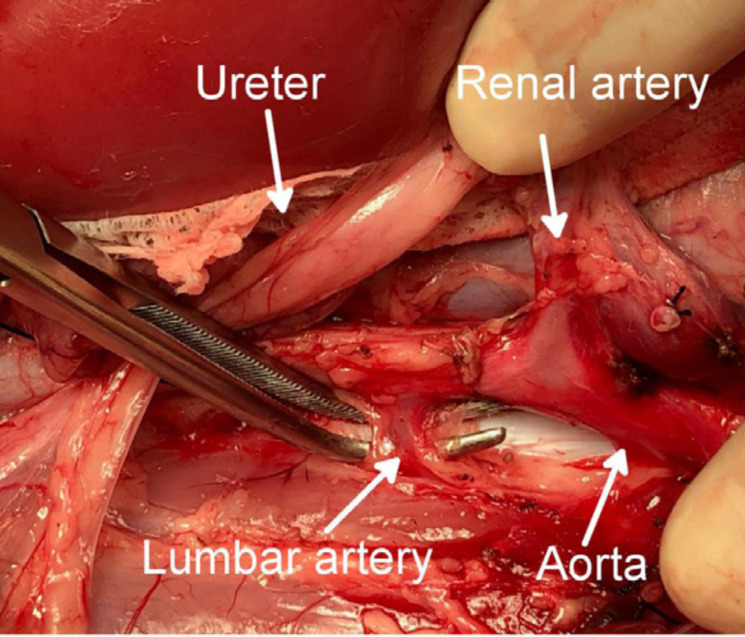
Ligation and division of the lumbar artery.

A full-length, midline, abdominal intraperitoneal incision was made from the xiphoid to the pubis; subsequent rightward mobilization of the intestine enabled the left kidney, renal artery, renal vein and aorta to be seen through the parietal peritoneum. After vertical incision of the parietal peritoneum around the renal hilum, the left kidney was mobilized by finger dissection and the perirenal tissue was dissected to identify the renal artery, vein and ureter. Ligating and dividing the lumbar vein diverging from the dorsum of the left renal vein was important to ensure that the left renal vein could be ventrally lifted and the aorta recognized behind the left kidney (Fig. 1); 6–7 cmof the aorta and inferior vena cava were dissected for cannulation (Fig. 2). The lumbar artery diverging from the dorsum of the aorta was ligated and divided to maximize the effectiveness of flushing the kidneys (Fig. 3).

**Figure 4:**
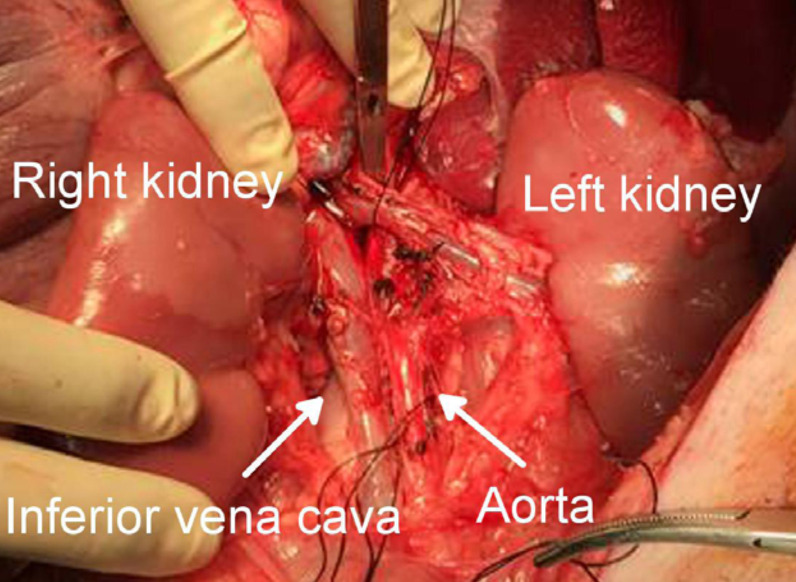
The recognition of both kidneys, the aorta and the inferior vena cava in the same field left side of the intestine.

Following leftward mobilization of the intestine, a vertical incision of the parietal peritoneum, mobilization of the right kidney and dissection of perirenal tissue were performed in the same manner. After confirming that the aorta and the inferior vena cava were exposed above the renal vessel bifurcations, mobilization of the right kidney to the left through the incision of the mesentery enabled both kidneys, the aorta and inferior vena cava to be recognized in the same field on the left side of the intestine (Fig. 4).

**Figure 5:**
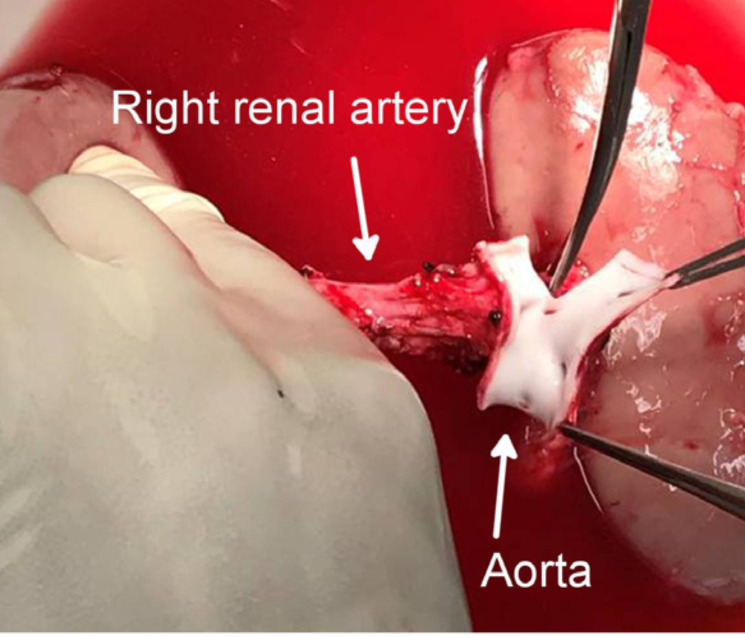
Separation of the right and left kidneys.

After the aorta was clamped above the renal artery bifurcation and ligated at the lower end of the dissection, the preservation solution (ET-Kyoto solution^9^) was infused via the catheter inserted above the ligation of the aorta. The inferior vena cava was ligated at the upper and lower ends of the dissection and cut below and above the ligation, respectively, for washing out. The aorta was cut above the clamp and below the ligation. The ureter was ligated and divided at the lower pole of the kidney; the right ureter was cut longer than the left. Both kidneys could be procured *en bloc* while perfusing the kidneys. After adding the perfusion at the back table, the right and left kidneys were separated and the renal arteries were trimmed, leaving the Carrel patch configuration about 10 mm in longitudinal length (Fig. 5). After removing both kidneys, phlebotomy in the thoracic cavity was performed for euthanasia and the surgical incision was closed.

### Recipient procedure

**Figure 6:**
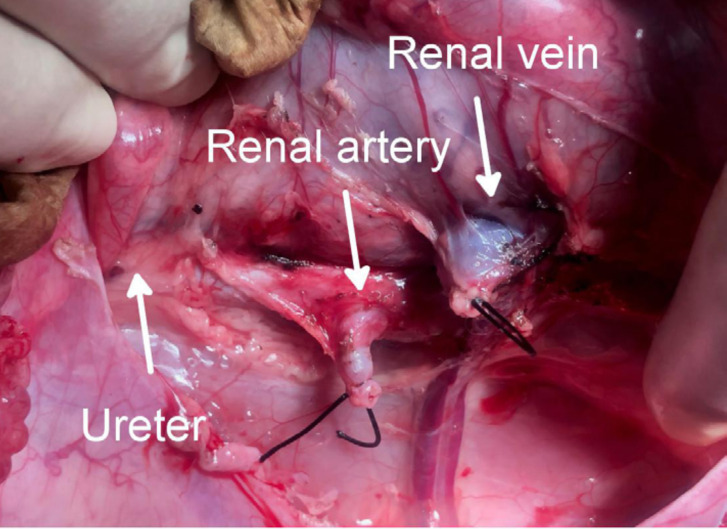
The aorta and the inferior vena cava after left nephrectomy.

**Figure 7:**
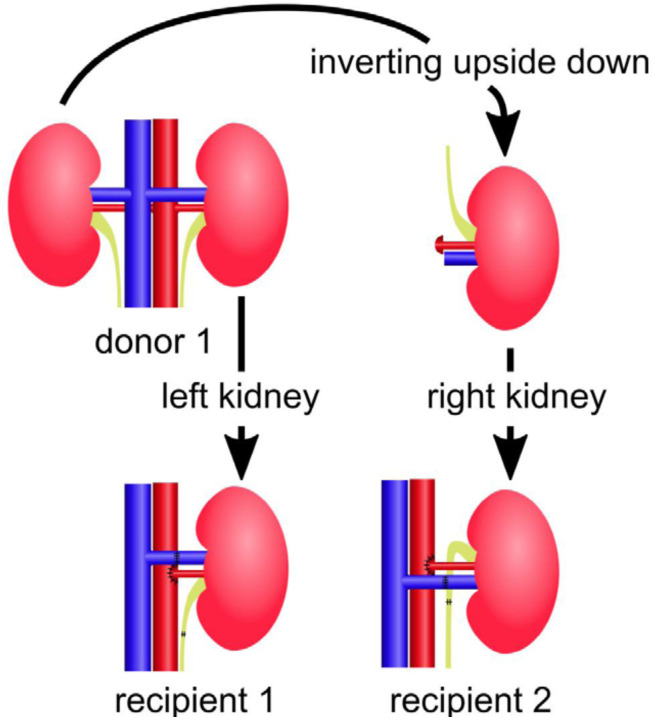
Inverting graft technique. When transplanting the right kidney into the left renal fossa, the graft was inverted upside down.

After laparotomy and dissection of the left kidney in the same manner as the donor, a left nephrectomy was performed (Fig. 6). When performing the orthotopic transplantation, ligation of the lumbar vein or dissection of the inferior vena cava was not required; therefore, the left renal vein was ligated and divided near the renal hilum to obtain a long margin. The aorta was minimally dissected, enabling it to be half-clamped after ligation and division of the renal artery (ligation of the lumbar artery was unnecessary). When the left kidney was the donor, the ureter was cut at the level of the lower pole of the kidney; however, when the right kidney was the donor, the ureter was cut at the level of the renal hilum while paying close attention to avoid impeding the blood supply. The graft was placed into the left renal fossa after nephrectomy. When the graft was the right kidney, it was inverted upside down, ensuring that the renal vein of the graft progressed anteriorly and anatomically natural vascular anastomosis could be performed; however, the ureter drew an upward loop and required a longer length (Fig. 7). After intravenous administration of 1000 units of heparin, the aorta was half-clamped with a Satinsky clamp. The root of the renal artery was cut and a slit incision of about 10 mm was made to create an anastomosis site; the grafted renal artery was anastomosed in an end-to-side fashion using a 5-0 polypropylene extraluminal continuous suture with two stay sutures. The renal artery was clamped with a bulldog clamp, and the aorta was declamped. The recipient’s renal vein was clamped with a bulldog clamp and ligation was resolved; the grafted renal vein was anastomosed end-to-end using a 6-0 polypropylene intraluminal continuous suture with two stay sutures^10^^,^ 11. The diameter of the renal vein was about 7 mm.

**Figure 8:**
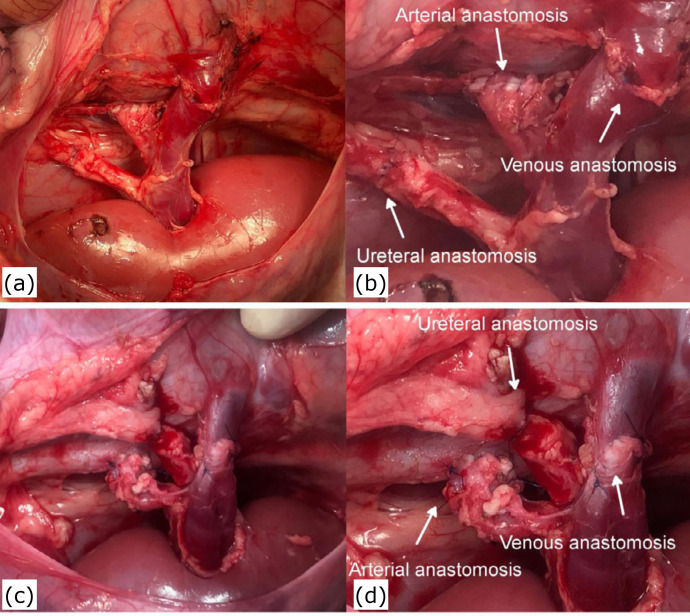
Kidneys after the anastomoses when the left kidney is transplanted into the left renal fossa (a, b) and when the right kidney is transplanted into the left renal fossa (c, d). The grafted renal artery with the Carrel patch configuration was about 10 mm in longitudinal length and anastomosed to the recipients’ aorta in a side-to-end fashion using a 5-0 polypropylene continuous suture. The grafted renal vein was 7 mm in diameter and anastomosed to the recipient’s renal vein in an end-to-end fashion using a 6-0 polypropylene continuous suture. The grafted ureter was about 2 mm in diameter and anastomosed to the recipient’s ureter in an end-to-end fashion using 6-0 polypropylene interrupted sutures.

The recipient’s ureter was carefully divided to avoid impeding the blood supply and anastomosed to the grafted ureter in an end-to-end fashion using 6-0 polypropylene interrupted sutures. The diameter of the ureter was about 2 mm and six stitches were performed. Figure 8 shows the kidneys after the anastomoses. After closure of the parietal peritoneum to fix the graft to the retroperitoneal space, the surgical incision was closed.

Surgical loupes of 1.5–2.5× magnification were used in vascular and ureteral anastomoses. The blood flows were confirmed with intraoperative ultrasonography when renal vessels were so narrow that the patency of the anastomoses was uncertain.

The recipients were euthanized at postoperative day 5 in a study evaluating the short-term outcomes without immunosuppressive agents, such as ischemia-reperfusion injury. The protocol for the study evaluating the long-term outcomes with immunosuppressive agents was previously reported^12^.

## Results and Discussion

In this paper, the focus was on transplantation techniques, aiming to demonstrate the surgical procedure of pig orthotopic kidney transplantation from one donor to two recipients via inverted grafting. This technique of inverting the graft upside down has been reported in human kidney transplantation to overcome a short renal vein obtained by laparoscopic right donor nephrectomy^13^^–^ 15; however, this paper is the first report to use this technique in a pig orthotopic kidney transplantation model. Using this technique, it would have been possible to develop a pig experimental model that is safe and has a high success rate, even for researchers in the middle of their training. In a previous research on chronic rejection, more than 30 procedures using this technique in a miniature pig model were performed^12^. No vascular complications occurred in cases in which this procedure was performed by an experienced surgeon. On the other hand, insufficient arterial flows were experienced in 20% of cases wherein the procedures were performed without Carrel patch technique (end-to-end anastomosis between donors’ and recipients’ renal arteries).

Transplantation from one donor to two recipients has some advantages, as reducing the number of pigs needed for experiments could reduce the animal burden, as well as the economic load. The outcomes of two recipients receiving kidneys from the same donor are easy to compare; however, the right and left kidneys are not anatomically identical. A surgical procedure considering the anatomical features of the donor kidney with the utmost care is needed.

Since the iliac vessels of pigs are too narrow to anastomose, the donor’s renal artery is generally anastomosed to the recipient’s aorta using Carrel patch technique, making the anastomosis easier and preventing stenosis^2^. There is no accepted theory regarding which side of the renal fossa is to be used for transplantation; therefore, the kidney was transplanted into the left renal fossa in our facility, considering the risk of the anastomosed renal artery oppressing the crossing inferior vena cava when transplanted into the right infrarenal fossa. The donor’s renal vein was anastomosed to recipient’s renal vein in an end-to-end fashion, as the graft was far from the inferior vena cava. Anatomically, the left kidney is naturally settled in the left renal fossa; however, when the right kidney is placed backwards into the left renal fossa, the renal vein of the graft progresses posteriorly and is anastomosed to the recipient’s left renal vein, located anteriorly. Kinking of the renal vein on the aorta and thrombosis are, therefore, a concern; thus, when the right kidney was transplanted into the left renal fossa, the graft was inverted upside down, not backwards. The anteroposterior relationship was, therefore, maintained and anatomically natural vascular anastomosis could be performed.

In human kidney transplantation, the anteroposterior relationship between the renal vessels can be corrected using an iliac vein transposition technique, in which the external iliac vein mobilizes laterally with respect to the external iliac artery^16^; however, the inferior vena cava or aorta could not be mobilized during pig orthotopic transplantation. An anatomically natural vascular anastomosis is important to prevent vascular complications, especially in pig kidney transplantations where postoperative resting is difficult and the kidneys hang due to quadrupedal walking. In addition, by using this technique, it was possible to perform a deeper anastomosis of the arteries prior to shallow anastomosis of the veins, making the arterial anastomosis easier.

By contrast, the technique of inverting the graft upside down has been reported in human kidney transplantation. Using this maneuver, the short renal vein obtained by laparoscopic right donor nephrectomy lies posteriorly, remaining closer to the external iliac vein and making venous anastomosis easy^13^^–^ 15. Instead, the ureter draws an upward loop and needs to be anastomosed to the recipient’s ureter, which does not increase urological complications such as hydronephrosis and urine leak^15^. Ureteroureterostomy could avoid using the long segment of the donor ureter which is reported to be a risk of ischemic necrosis, as the middle segment of the ureter receives blood supply from the common iliac artery and its branches^17^.

## Conclusion

The surgical procedure of pig orthotopic kidney transplantation from one donor to two recipients was demonstrated. When the right kidney was transplanted into the left renal fossa, an anatomically natural vascular anastomosis could be performed via inverted grafting.
